# Comparison of within 7 Day All-Cause Mortality among HDU Patients with Modified Early Warning Score of ≥5 with those with Score of <5

**DOI:** 10.12669/pjms.37.2.2832

**Published:** 2021

**Authors:** Majid Ahmed Shaikh, Avinash Punshi, Mohan Lal Talreja, Tazeen Rasheed, Nimrah Bader, Bader Faiyaz Zuberi

**Affiliations:** 1 Majid Ahmed Shaikh, FCPS, Department of Medicine, Dow Medical College, Dow University of Health Sciences, Karachi, Pakistan; 2 Avinash Punshi, FCPS, Department of Medicine, Dow Medical College, Dow University of Health Sciences, Karachi, Pakistan; 3 Mohan Lal Talreja, MRCP, Department of Medicine, Dow Medical College, Dow University of Health Sciences, Karachi, Pakistan; 4 Tazeen Rasheed, FCPS, Department of Medicine, Dow Medical College, Dow University of Health Sciences, Karachi, Pakistan; 5 Nimrah Bader, Department of Internal Medicine University of Oklahoma Health Sciences Center, Oklahoma City, OK, USA; 6 Bader Faiyaz Zuberi, FCPS, Department of Medicine, Dow Medical College, Dow University of Health Sciences, Karachi, Pakistan

**Keywords:** MEWS Score, Mortality, ROC

## Abstract

**Objective:**

To compare 7-Day All-Cause Mortality among HDU Patients with Modified Early Warning Score of ≥5 with Those with Score of <5.

**Methods:**

All patients of age more than 18 years, of either gender admitted in HDU of Medical Unit-II, CHK between September 2019 to February 2020 were included. MEWS was calculated for each patient at time of admission. Patients with MEWS score of ≥5 were allocated to Group-A and those with score of <5 were allocated to Group-B. Patients were followed for seven days and outcome status of alive, expired or discharged was noted.

**Results:**

Total of 336 patients were selected out of which 168 patients was inducted in Group-A and 168 patients in Group-B. MEWS Score in patients who expired was significantly higher (Mdn=11) than in those who survived (Mdn=4), p <.001. 7-day mortality in Group-A was 62 (39.9%) while in Group-B was 40 (23.8%). ROC was plotted of MEWS Score for mortality, it showed significant area under curve of 68.4% (p <.001, 95% CI = .62 to .75). MEWS Score of 3.5 showed sensitivity of 89.2% and specificity of 65%.

**Conclusion:**

Our results show that MEWS has a positive trend to predict mortality. MEWS score of 3.5 is suggested cut off based on ROC in our study.

## INTRODUCTION

Early recognition and immediate resuscitation are fundamentals of successful management of all critically ill patients if they are suffering from infection and sepsis, malnutrition, AIDS, trauma, diabetes mellitus, drug overdose, and poisoning.^1^ In most, seriously ill patients, initial diagnosis may not be clear and immediate objective is to save the life and reverse or prevent vital organ damage e.g. brain, lungs, liver and kidneys.^2^ A rapid identifying, low cost method called as Modified Early Warning Score (MEWS) that utilize easy to measure physiological parameters such as vital signs and level of consciousness can be used to identify critical illness, facilitate early intervention and predict mortality.^3^,^4^ MEWS values range from 0 to maximum 14, higher scores mean greater hemodynamic instability.^5^ A score of five or more identifies a patient to be critically ill and is associated with increased risk of Intensive care unit (ICU) admission and death.^6^ MEWS is a reliable screening tool to identify critically ill patients early and to act timely to improve outcomes in health care and prevent adverse events like cardiac arrest, renal failure.^7^ A study performed in Uganda on sepsis patients demonstrated that early IV fluid and antibiotic therapy together with vital sign monitoring was associated with lowered 30-day mortality.^8^

Early categorization of critically ill patients by calculating MEWS score in hospitals may give a time window for appropriate steps. If a patient is suffering from sepsis, timely intravenous fluids, early antibiotics and monitoring in a low resource country like Pakistan, may have a great impact.^9^,^10^ Therefore, the current research is planned to early identify critically ill patients by applying MEWS and reducing the mortality by providing early management and taking appropriate life saving measures.

## METHODS

This observational longitudinal study was conducted at Dr Ruth K. M. Pfau, Civil Hospital Karachi between September 2019 to February 2020. Non-probability consecutive sampling was used for selection of patients. Approval was taken from the Institutional Review Board of Dow University of Health Sciences Ref: no. IRB-1262/DUHS/Approval/2019 Dated 01^st^ August 2019.

### Inclusion criteria

All patients of age more than 18 years, of either gender admitted in HDU of Medical Unit-II, CHK were included. Patients with neurosurgical trauma, orthopaedic or general surgery trauma and obstetrics patients were clinically assessed and excluded.

### Sample Size

A sample of 168 from the positive group and 168 from the negative group achieves 80% power to detect a difference of 0.05 between the area under the ROC curve (AUC) under the null hypothesis of 0.90 and an AUC under the alternative hypothesis of 0.85 using a two-sided z-test at a significance level of 0.05. Sample size calculation was done by PASS 2019 software.

A written informed consent was taken. MEWS was calculated for each patient at time of initial assessment on admission. Patients were segregated into two groups on basis of MEWS. Patients with score of ≥5 were allocated to Group-A and those with score of <5 were allocated to Group-B. Patients were followed for 7 days and outcome status of survive, expired or discharged was noted. Patients discharged before 7^th^ day were assumed as survive for analysis.

Categorical data was presented as frequency and percentage, e.g., gender & mortality. Quantitative data was presented as mean with standard deviation, e.g., total number of patients presenting to medical emergency & patients’ MEWS. Quantitative data was tested for normal distribution by Shapiro-Wilk (SW) Test. The results SW test will determine whether parametric or non-parametric tests will be used for analysis. Effect modifiers like age & gender was controlled through risk stratification. Post stratification χ^2^ test was used for qualitative variables. Frequency of mortality between Group A & B was compared using Chi-square test. ROC curves were plotted for mortality & sensitivity of MEWS was calculated in our population. P-value of ≤ 0.05 was considered as level of significance. Data was analysed using software SPSS version 26. Modified Early Warning Score (MEWS) is given in Table-I.^9^

**Table-I:** Modified Early Warning Score.

Score	+3	+2	+1	0	+1	+2	+3
Systolic blood pressure (mmHg)	<70	71-80	81-100	101-199		>200	
Pulse rate (beats/min)		<40	41 -50	51-100	101-110	111-129	>130
Respiratory rate (breaths/minute)		<9		9-14	15-20	21-29	>30
Temperature (^o^C/^ o^F)	<35/95			35-38.4/95-101.1		>38.5/101.3	
AVPU score				Alert	Reaction to voice	Reaction to pain	Unresponsive

## RESULTS

Total of 336 patients were selected out of which 168 patients with MEWS Score of ≥5 was inducted in Group-A and 168 patients with MEWS Score of <5 was inducted in Group-B. The distribution of variables of age, gender, time to present to ICU from onset of symptoms and outcome at day seven, were tested for normal distribution by Kolmogorov-Smirnov Test and all the variables were found not normally distributed, hence non-parametric test were used for analysis. The descriptive statistics are reported as median (Mdn) ± standard deviation (SD) are given in Table-I.

**Fig.1:**
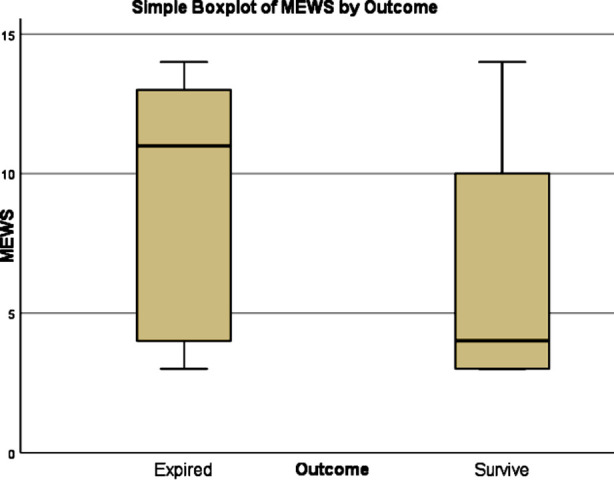
Box plot of MEWS Score with Outcome.

Comparison of quantitative variables among groups was done by Mann-Whitney Test. Age in Group-A was significantly greater (Mdn=55) than that in Group-B (Mdn=48), U=9355, p <.001. Time to present from onset of symptoms to hospital was not statistically significant between Group-A (Mdn=7) and Group-B (Mdn=6.5), U=13644, p=.598. MEWS Score in Group-A was significantly greater (Mdn=11) than that in Group-B (Mdn=3), U=0, p <.001. MEWS Score was also assessed with outcome and was found the score in patients who expired was significantly higher (Mdn=11) than in those who survived (Mdn=4), U=7552.5, p <.001. Box Plot of MEWS Score with Outcome is given in Figure-1. Mortality in both groups was compared by non-parametric (NPAR) χ^2^ test. 7-day mortality in Group-A was 62 (39.9%) while in Group-B was 40 (23.8%). Mortality in Group-A was significantly higher. χ^2^ (df = 1, N = 336) = 51.9, p <.001.

**Fig.2:**
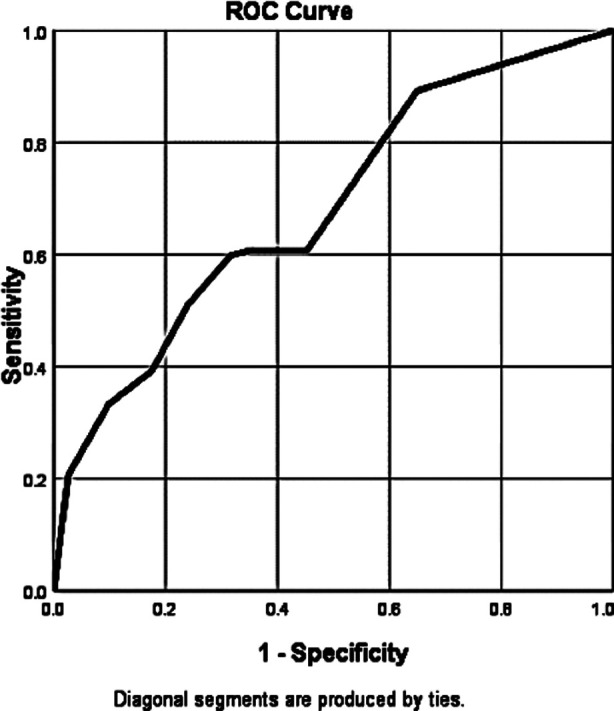
ROC Plot of MEWS Score for Mortality.

ROC was plotted of MEWS Score for mortality in outcome and given in Figure 2. It showed significant area under curve of 68.4% (p <.001, 95% CI = .62 to .75). MEWS Score of 3.5 showed sensitivity of 89.2% and specificity of 65%.

## DISCUSSION

Our study showed significantly higher 7-day mortality in patients with MEWS Score of ≥5 (39.9%) on admission as compared to those with <5 (23.8%). This correlates with other studies which states that low scores correlate with low mortality.^12^,^13^ This is first study from our area and have stressed the need of early assessment and proper resource allocation for better outcome. Similar observations were also made by Kia A et al.^10^ Suboptimal hospital treatment is common in the period before Intensive Care Unit admission and is associated with increased Intensive Care Unit and hospital mortality. Many hospital deaths are potentially preventable. Catastrophic deterioration of patients in hospital is usually preceded by deterioration in a number of physiological parameters.^11^ Several studies have shown that abnormal physiological values are usually charted in the hours before patients suffer an in-hospital cardiorespiratory arrest. The MEWS is a tool for bedside evaluation based on five physiological parameters: systolic blood pressure, respiratory rate, pulse rate, temperature and AVPU score. MEWS has a score ranging from 0 (lowest) to three (highest) for all its 5 parameters. The total score of all five parameters represents the MEWS.^12^ Lower values of MEWS predict better outcomes and higher values are considered predictive of poor outcomes.^12^

**Table-II:** Comparison of age, time to present, MEWS Score and mortality between two groups.

	*Group-A*	*Group-B*	Sig*
Age	55.0 ±10.0	48.0 ±12.0	<.001*β
Time to Present	7.0 ±3.8	6.5 ±3.2	.598β
MEWS Score	11.0 ±2.3	3.0 ±0.5	<.001β
Mortality	62 (39.9%)	40 (23.8%)	.013†

* Significant Value ≤.05,

β Mann-Whitney Test,

† C^2^ Test.

In our study we aimed to compare the 7-Day all-cause mortality among High Dependency Unit (HDU) patients admitted in our ward with MEWS of ≥5 with those with score of < 5. The results of our study showed that patients with MEWS >5 had higher mortality as compared to patients with MEWS <5. Our results were similar to a study by Subbe C et al. in which they applied MEWS to 709 medical emergency patients and their results showed that Scores of five or greater were associated with increased risk of death (OR 5.4, 95%CI 2.8–10.7), ICU admission (OR 10.9, 95%CI 2.2–55.6) or HDU admission (OR 3.3, 95%CI 1.2–9.2).^11^ In another study conducted by Ho Le O et al. in which MEWS was applied to critically ill patients presenting to a tertiary emergency department and it was reported that Mews <4 was associated with poor prognosis.^12^ Reini K et al reported that patients with a MEWS of at least 6 had significantly higher mortality in the ICU than those with a MEWS <6 and that MEWS of at least six on admission was also associated with high 30-day mortality and increased length of stay in the ICU.^13^

Gardner-Thorpe J et al. in their study used a cut off MEWS value of four and reported that MEWS with a threshold of 4 or more had a 75% sensitivity and 83% specificity for patients who needed to be transfer to ICU or HDU.^14^ In our study the ROC showed the best cut-off values for mortality at 3.5 which is lower than all the studies quoted above. In our study the sensitivity and specificity of 65% and 89.2% respectively of MEWS Score of 3.5. This means that in our population the risk of mortality is higher even at lower scores of MEWS. It has been reported at for every one point increase in MEWS there in 33% increase in chance of hospitalization.^15^ In view of findings of our study we should consider urgency if MEWS is >3.5, take urgent decisions and measures to prevent it and provide early interventions.^16^

### Limitations of the study

Although our study was adequately powered and had an appropriate sample size, only limitation of the study was being a single centre study. But this should generate interest in subject which is not under the radar of medical community at present, due to difficulty in carrying out a study in potentially extremely sick patients requiring urgent care.

## CONCLUSION

Our results show that MEWS has a positive trend to predict transfer to ICU and in-hospital death. MEWS score of 3.5 is suggested cut off based on ROC in our study. Thus, early categorization of critically ill patients by calculating MEWS score in hospitals may give a time window for appropriate steps to be taken.

### Authors’ Contribution:

**MAS:** Study Conception & data integrity.

**AP:** Data collection, initial manuscript writing & data integrity.

**ML:** Manuscript review and statistical analysis & data integrity.

**BFZ:** Final corrections and approval of manuscript & data integrity.

**TR:** Data collection, initial manuscript writing & data integrity.

**NB:** Manuscript writing and statistical analysis.

All authors are responsible and accountable for the accuracy and integrity of the work.
